# Prevalence and determinants of cigarette smoking and smoking frequency among people with tuberculosis in Lesotho: Evidence from a nationwide cross-sectional survey

**DOI:** 10.1371/journal.pone.0335002

**Published:** 2026-06-05

**Authors:** Calista Ogechukwu Dozie-Nwakile, Alphonsus Ogbonna Ogbuabor, Nwanneka Chidinma Ghasi, Daniel Chukwuemeka Ogbuabor

**Affiliations:** 1 Department of Medical Laboratory Sciences, Faculty of Health Sciences and Technology, University of Nigeria‌‌, Enugu Campus, Enugu, Nigeria; 2 Department of Medical Laboratory Sciences, School of Allied Health Sciences, Kampala International University, Kampala‌‌, Uganda; 3 Department of Management, Faculty of Business Administration, University of Nigeria, Enugu Campus, Enugu, Nigeria; 4 Department of Health Administration and Management, Faculty of Health Sciences and Technology, University of Nigeria, Enugu, Nigeria; Debre Tabor University, ETHIOPIA

## Abstract

**Background:**

Low- and middle-income countries face a significant dual burden of tuberculosis (TB) and cigarette smoking. Nevertheless, studies examining the prevalence and determinants of smoking status and frequency among people with TB (PWTB) are scarce in sub-Saharan Africa, including Lesotho. Therefore, this study evaluated the prevalence and determinants of smoking status and frequency among people with TB in Lesotho.

**Methods:**

Data on people with TB from the 2023 Lesotho Demographic and Health Survey were used in this study (n = 326). The data were adjusted for sampling weight, stratification, and cluster sampling design. The outcome variables were smoking status and smoking frequency (daily smoking and occasional smoking). The independent variables included socio-demographic factors, household characteristics, alcohol use, TB-related stigma, and mental health status. We evaluated the association between outcome and predictor variables using Pearson’s chi-squared test and complex sample logistics regression. Statistical significance was set at a p-value < 0.05.

**Results:**

The prevalence of cigarette smoking among PWTB is 23.8%. The prevalence of daily and occasional smoking among PWTB is 17.3% and 6.5%, respectively. Being male (AOR = 107.48, 95%CI:22.65-510.03, p < 0.001), no media exposure (AOR = 3.65, 95%CI:1.43-9.30, p = 0.007), and alcohol use (AOR = 6.98, 95%CI:2.21-22.05, p = 0.001) increased the odds of smoking among PWTB. Being male (AOR = 211.28, 95%CI:25.86-1725.91, p < 0.001), no media exposure (AOR = 3.95, 95%CI:1.27-12.26, p = 0.018), and alcohol use (AOR = 15.10, 95%CI:3.36-67.89, p < 0.001) increased the odds of daily smoking among PWTB. Being male (AOR = 39.98, 95%CI:4.76-335.88, p = 0.001) increased the odds of occasional smoking among PWTB. Depression did not significantly influence smoking status of people with TB.

**Conclusion:**

The prevalence of smoking among PWTB is high in Lesotho. Our findings underscore the importance of gender-sensitive tobacco use prevention and control interventions among PWTB, an inclusive and adequately funded long-term plan for best-practice anti-tobacco media campaign, and integrating management of substance use disorder into TB control policy and routine TB care in Lesotho.

## Background

Low- and middle-income countries (LMICs) face a significant dual burden of tuberculosis (TB) and cigarette smoking [1]. In 2023, approximately 10.8 million people globally were affected by TB, leading to an estimated 1.25 million deaths [2]. Despite a decline in incidence rates, sub-Saharan Africa (SSA) continues to host over 50% of the countries with a high burden of TB, including Lesotho [2]. Although there has been a 15% reduction in TB incidence, Lesotho’s rate remains high at 664 cases per 100,000 people [2]. Lesotho has the highest multidrug-resistant TB burden connected to smoking, with age-standardized disability-adjusted life-year (DALY) rate and age-standardized DALYs rates per 100,000 people 4.45 (95%UI, 0.99–13.52) and 156.12 (95%UI, 34.26–471.88) [3]. Equally, more than 80% of the 1.3 billion smokers worldwide live in low- and middle-income countries, contributing to over eight million deaths each year from tobacco-related causes [4]. In SSA, the prevalence of cigarette smoking stands at 7.1%, which is significantly lower than Lesotho’s 18.3% [4]. Smoking accounts for more than 20% of global TB incidence and about 1,400 TB cases in Lesotho [2]. Current smokers are more likely to have both symptomatic and subclinical TB [5]. Ever-smokers, current smokers, and past smokers have an elevated risk of TB in comparison to never-smokers [6]. Consequently, the End TB strategy emphasizes the importance of TB screening for smokers and incorporates smoking cessation into standard TB care practices [7].

Studies conducted in various settings have shown that tobacco smoking among people with tuberculosis (TB) is significantly associated with several adverse health outcomes. These include longer diagnostic delays [8], a higher likelihood of positive sputum smears and cavitary TB [8–11], prolonged sputum conversion time [12,13], increased rates of TB relapse or recurrence [1,14–16], greater chances of drug-resistant TB, poor adherence to treatment [17,18], and higher rates of loss to follow-up [17–20], treatment failure [14,15,17–20], and death [1,14,15,17,19,20]. In high-burden TB countries, smoking contributes to 15% of TB-related mortality [21]. Furthermore, a systematic review revealed that smoking increases the likelihood of poor treatment outcomes by 51% globally and by 74% in LMICs [22]. However, a West African study found no significant differences in disease severity at the time of diagnosis and no notable differences in treatment outcomes, which are attributable to confounding socioeconomic factors [23].

The prevalence of smoking among TB patients varies widely. In SSA, the prevalence of smoking among people with TB ranges from 15.2% to 26% in Uganda [24,25], whereas South Africa exhibits alarmingly high rates between 56% and 82% [26–28]. Additional studies indicate prevalence rates of 16.2% in Ethiopia [29], 25% in Botswana [30], and 30% in Gabon [8]. These findings highlight a need for targeted smoking cessation interventions for individuals diagnosed with TB in different settings. A comprehensive understanding of the social determinants of smoking among people with TB is essential for developing and implementing focused smoking cessation interventions for those most at risk. Existing literature suggests that age [31], male gender [11,17,28,30–33], urban residency [17], lower education [28], employment status [17], alcohol use [11,17,33,34], depression [30], and financial difficulties [32] are risk factors for smoking among people with TB.

There is a paucity of studies examining the prevalence and determinants of smoking among people with TB globally, particularly in SSA. Most African studies utilize facility-based surveys that are not nationally representative or do not focus on social determinants [8,13,23–25,27–29,31,33]. In Lesotho, existing health system research on TB focus on evaluating determinants of treatment outcome, understanding the barriers to TB care, and knowledge and attitude towards TB [35–38]. Lesotho’s National Tuberculosis Strategic Plan 2018–2022 did not prioritize smoking as a critical social determinant of TB [39]. To our knowledge, no published study has assessed the prevalence and predictors of smoking among people with TB in Lesotho despite having the highest incidence of TB in SSA [2], and a high prevalence of smoking in the general population [4]. Therefore, our study fills an important gap in the literature by presenting new evidence regarding the prevalence and determinants of smoking among people with TB in Lesotho utilizing a nationally representative population-based survey. Such evidence would be helpful to TB policymakers, program managers, and service providers in prioritizing smoking in TB strategic plans and integrating smoking cessation interventions into routine TB care and prevention strategies.

## Methods

### Study setting

Lesotho is a small lower-middle-income country with a population of 2.3 million and a gross domestic product (GDP) per capita of USD1,300. Almost two-thirds (67%) of the population lives in rural areas, and females account for 51% of the population. There are high levels of inequality in Lesotho, with a Gini coefficient of 44.6%, and 24% of people live in extreme poverty [40]. Lesotho has ten administrative districts. Each district is subdivided into constituencies, and each constituency into community councils. The districts serve as TB programme coordinating and reporting units under the leadership of the ten District Health Management Teams. The country achieved the 2020 milestone of the End TB strategy (a 20% reduction compared with the 2015 baseline). Nevertheless, the TB programme has a 68% case identification gap and a 76% treatment success rate.

### Study design‌‌

We analysed secondary data from the 2023−24 Lesotho Demographic and Health Survey (LDHS). The primary study used a cross-sectional, household survey design. We accessed the LDHS data set upon request through the ICF Macro DHS data management portal.

### Sampling strategy

The primary study calculated the sample size of households (n = 10,000) using information obtained from the 2014 LDHS. The sample calculations used information obtained from the 2014 LDHS: the average number of women age 15–49 per household was 0.816 in urban areas and 0.687 in rural areas, the average number of men age 15–59 per household was 0.683 in urban areas and 0.663 in rural areas, the household completion rate was 94.6%, the response rate among women age 15–49 was 97.1%, and the response rate among menage 15–59 was 94%.

**Fig 1:**
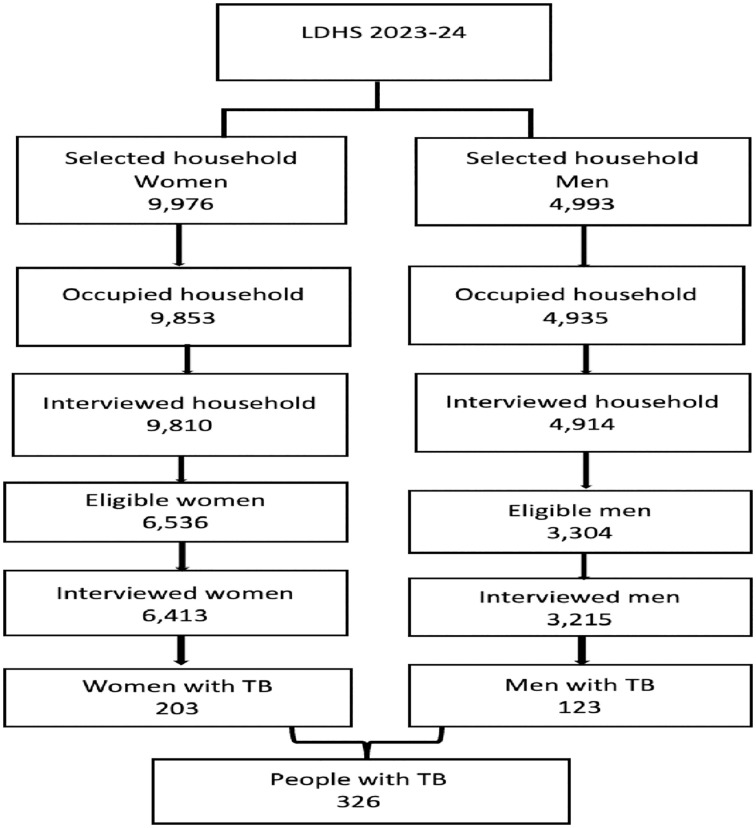
Flowchart for the sampling.

The 2023−24 LDHS used a two-stage stratified sampling technique to select the households. The sampling frame consisted of households listed in Lesotho’s 2016 Population and Housing Census (PHC) conducted by the Lesotho Bureau of Statistics (BoS). The primary sampling unit (PSU) is a cluster of enumeration areas. An enumeration area (EA) is a geographical area, usually a city block in urban areas or a village in rural areas, with an adequate number of households. Each district was stratified into urban and rural areas. In the first stage, the study selected 400 (176 urban and 224 rural) EAs from the sampling strata with probability proportional to EA size. The second stage’s sampling frame is the household list in each EA. In the second stage, 25 households were selected from every EA through equal probability systematic sampling, resulting in a total sample size of about 10,000 households (4,400 urban and 5,600 rural households). However, the study selected 9,976 households for the Women’s Questionnaire and 4,993 (from a subsample of half of the women’s households) for the Men’s Questionnaire (Fig 1). No replacements and no changes to the preselected households were allowed in the implementation stages to prevent bias.

### Data collection

The data collection for the primary study took place over 3 months from 27 November 2023–29 February 2024 across the 10 districts of Lesotho. All women age 15–49 (n = 6,536) and all men age 15–59 (n = 3,304) in the subsample who were usual residents of the sampled households or stayed in the households on the night before the interview were eligible for interviews. Data were successfully collected from 6,413 women and 3,214 men, representing 98.1% and 97.3% of eligible women and men, respectively. The survey data were collected using tablet computers running the Android operating system and Census and Survey Processing System (CSPro) software. The data collectors comprised 15 field teams, each consisting of one team supervisor, three or four female interviewers, and one to three male interviewers. Each interviewer entered the answers to the survey questions into the tablets. Supervisors downloaded interview data to their tablet, checked it for completeness, and monitored fieldwork progress. In the current study, we extracted and analysed the TB patient data between 3 February and 24 March 2025.

## Variables

### Outcome variable

The outcome variables were smoking status and smoking frequency at the time of the survey. In this study, smoking cigarettes included smoking combustible cigarettes such as manufactured cigarettes and hand-rolled cigarettes [41], but excluded cigars, cheroots, cigarillos, hookah, kreteks, and e-cigarettes. The study assessed current cigarette smoking using the specific question “Do you currently smoke cigarettes?” Respondents who answered “yes” to the question were classified as current cigarette smokers, while those who answered “no” were classified as women who do not currently smoke. Furthermore, the smokers were asked whether they smoked every day or only on some days. We recoded the smoking frequency into a binary variable: daily (every day) and occasional (some days) smoking.

### Independent variables

We selected the independent variables based on previous studies on smoking in Africa and the data availability in the DHS database. The factors included in this study are gender (male and female), age (15–19, 20–29, 30–39, and ≥40 years), marital status (Never in a union, married/living with a partner, and divorced/separated/widowed), district, place of residence (urban and rural), highest education (no education, primary, secondary, and higher), religion (Catholic, Protestants, Pentecostal/other Christians, and Others), Ethnicity (Basotho or other), sex of household head (female and male), household size (≤5 and >5), media exposure (No, low, and high), ownership of mobile phone (yes, no), wealth index (poorest, poor, moderate, rich, richest), occupation (not working, professional/technical/managerial, clerical/sales/services, agricultural, and manual), employment (unemployed and employed), total children (no child, one, 2–4, and >4), health insurance ownership (No and Yes), alcohol use (No and Yes), wife beating (No and yes), and TB-related stigma (No stigma and Yes). Wife-beating attitude was measured using five variables describing respondents’ attitudes towards domestic violence, including whether beating was justified if the wife goes out without telling her husband, neglects the children, argues with her husband, refuses sex, and burns food [42]. People who answered ‘Yes’ and ‘Don’t know’ were coded as 1, while those who responded ‘No’ were coded as 0. Respondents were categorised into good gender attitude if they answered ‘No’ to all five variables, and poor gender attitude if they answered ‘Yes’ or ‘Don’t know’ to any of the five questions.

We derived a composite measure of TB-related stigma using three specific questions: whether a respondent believes TB is curable, keeps secret if a family member has TB, and is willing to work with someone with TB. Individuals who answered “yes” to questions regarding tuberculosis (TB) treatment options or their willingness to work with someone with TB were coded as ‘1’. In contrast, those who answered “no” or “don’t know/not sure/depends” were recoded as ‘0’. For the question regarding keeping TB a secret, respondents who answered “no” or “don’t know/not sure/depends” were recoded as ‘1’, while those who answered “yes” were recoded as ‘0’. We summed the scores after completing the recoding process. We classified individuals who obtained a total score of 3 as having “no stigma,” and those with scores below three as having “stigma.”

The 2023−24 LDHS screened respondents’ mental health using the Patient Health Questionnaire, or PHQ-9 (Kroenke & Spitzer, 2002). The sum of the scores on each of the nine items forms the PHQ-9 score. Each symptom in the PHQ-9 is assigned a score of 0, 1, 2, or 3 depending on how frequently the respondent reported experiencing the symptom in the 2 weeks preceding the survey: 0 – Never, 1 – Rarely, 2 – Often, and 3 – Always. PHQ-9 scores range from a minimum of 0 to a maximum of 27. Higher scores reflect more severe symptoms of depression. A PHQ score of 0–4 indicates minimal symptoms or no symptoms, while a score of 5–9 is considered mild, 10–14 is moderate, 15–19 is considered moderately severe, and 20–27 is considered severe. In our analysis, we recoded people with minimal or no symptoms to ‘0’, mild to ‘1’, moderate to moderately severe, and severe to ‘2’.

### Statistical analysis

We analysed the data using Statistical Package for Social Sciences (SPSS) version 20 (IBM Corp., Armonk, NY). Before analysis, we adjusted the data for sampling weights, stratification, and multistage sampling to account for the variation in sample allocation to the districts and provide representative population estimates. Individual sample weights were generated by dividing (v005 and mv005) by 1,000,000 before analysis to approximate the number of cases. We checked for the multicollinearity of the independent variables using variance inflation factors (VIF), and they ranged from 1.17 to 2.08. We reported descriptive statistics using frequencies, population estimates and percentages (weighted). We assessed the association between the outcome variables (smoking status and frequency) and the independent variables using the Chi-square test. All variables whose univariable test had a p-value of less than 0.05 were included in the multivariable complex sample logistic regression model to determine the adjusted effect of each predictor variable on smoking status or frequency. The results of regression analysis were presented by crude/unadjusted odds ratio (COR) and adjusted odds ratio (AOR) with 95% confidence intervals (CIs), and p-values of less than 0.05 were considered significant. The study adopted p-values less than 0.05 in selecting variables to improve the focus, clarity, and predictive accuracy of the models. We tested the model fit using McFadden’s R-squared because it compares the likelihood-ratio of the current model to a model without any covariates and represents the amount of variation explained by the current model [43]. The McFadden test statistic for smoking status and smoking frequency was 0.55 and 0.47, respectively, indicating a good model fit [43].

### Ethical consideration

We did not obtain further ethical approval as this was a secondary data analysis. The 2023–24 LDHS protocol received clearance from the ICF Institutional Review Board ethics committee and the Lesotho Ministry of Health Research and Ethics Committee. Additionally, interviewers obtained informed consent from participants before conducting interviews.

## Results

### Basic characteristics of people with TB

Table 1 shows the basic characteristics of the respondents. Over 60% of the respondents were females, married or living with a partner, and employed. About 77% of the respondents lived in three of the country’s nine districts (Maseru, Leribe, and Berea districts). Most respondents are Christians, have at least basic education, hail from Basotho tribe, and own a mobile phone. Most household have five or less people. Almost 35% of households are poor. Health insurance coverage is low. Approximately 65% of the respondents use alcohol.

**Table 1:** Basic characteristics of people with TB in Lesotho, 2023−24 (N = 326).

		Frequency (n)	Percent (%)
Gender	Male	123	37.8
	Female	203	62.2
Age group	15-19	8	2.5
	20-29	29	9.0
	30-39	88	27.0
	40 and above	201	61.6
Currently/formerly/never in union	Never in union	44	13.5
	Currently in union/living with a woman	209	64.1
	Formerly in union/living with a woman	73	22.4
District	Butha-Buthe	13	4.0
	Leribe	58	17.9
	Berea	49	15.2
	Maseru	143	43.9
	Mafeteng	17	5.1
	Mohale’s Hoek	19	5.8
	Quthing	8	2.4
	Qacha’s Nek	6	1.8
	Mokhotlong	2	0.7
	Thaba-Tseka	10	3.1
Type of place of residence	Urban	173	53.2
	Rural	153	46.8
Educational level	No education	15	4.8
	Primary	125	38.3
	Secondary	147	45.1
	Higher	38	11.8
Religion	Roman Catholic	140	42.8
	Protestant	83	25.5
	Pentecostal/other christians	91	27.9
	Other religons	12	3.8
Ethnicity	Basotho	315	96.6
	Others	11	3.4
Sex of household head	Male	218	66.9
	Female	108	33.1
Household size	≤5 persons	234	71.8
	>5 persons	92	28.2
Media exposure	No	42	12.9
	Yes	284	87.1
Owns a mobile telephone	No	63	19.3
	Yes	263	80.7
Wealth index for urban/rural	Poorest	54	16.6
	Poorer	60	18.3
	Middle	70	21.5
	Richer	58	17.9
	Richest	84	25.7
Occupation	Not working	70	21.6
	Professional/Technical/Managerial	12	3.7
	Clerical/Sales/Services	76	23.2
	Agricultural workers	24	7.5
	Manual workers	144	44.1
Currently working	No	122	37.5
	Yes	204	62.5
Total Children	No child	45	13.9
	One child	79	24.1
	2-4 children	162	49.8
	>4 children	40	12.1
Covered by health insurance	No	284	87.2
	Yes	42	12.8
Alcohol use	Never	113	34.7
	Yes	213	65.3
Wife beating	No	265	81.5
	Yes	60	18.5
TB-related Stigma	No stigma	277	85.0
	Stigma	49	15.0
Depression	Missing data	102	31.2
	No depression	160	49.0
	Yes, depression	51	19.8

### Prevalence of smoking among people with TB

**Fig 2:**
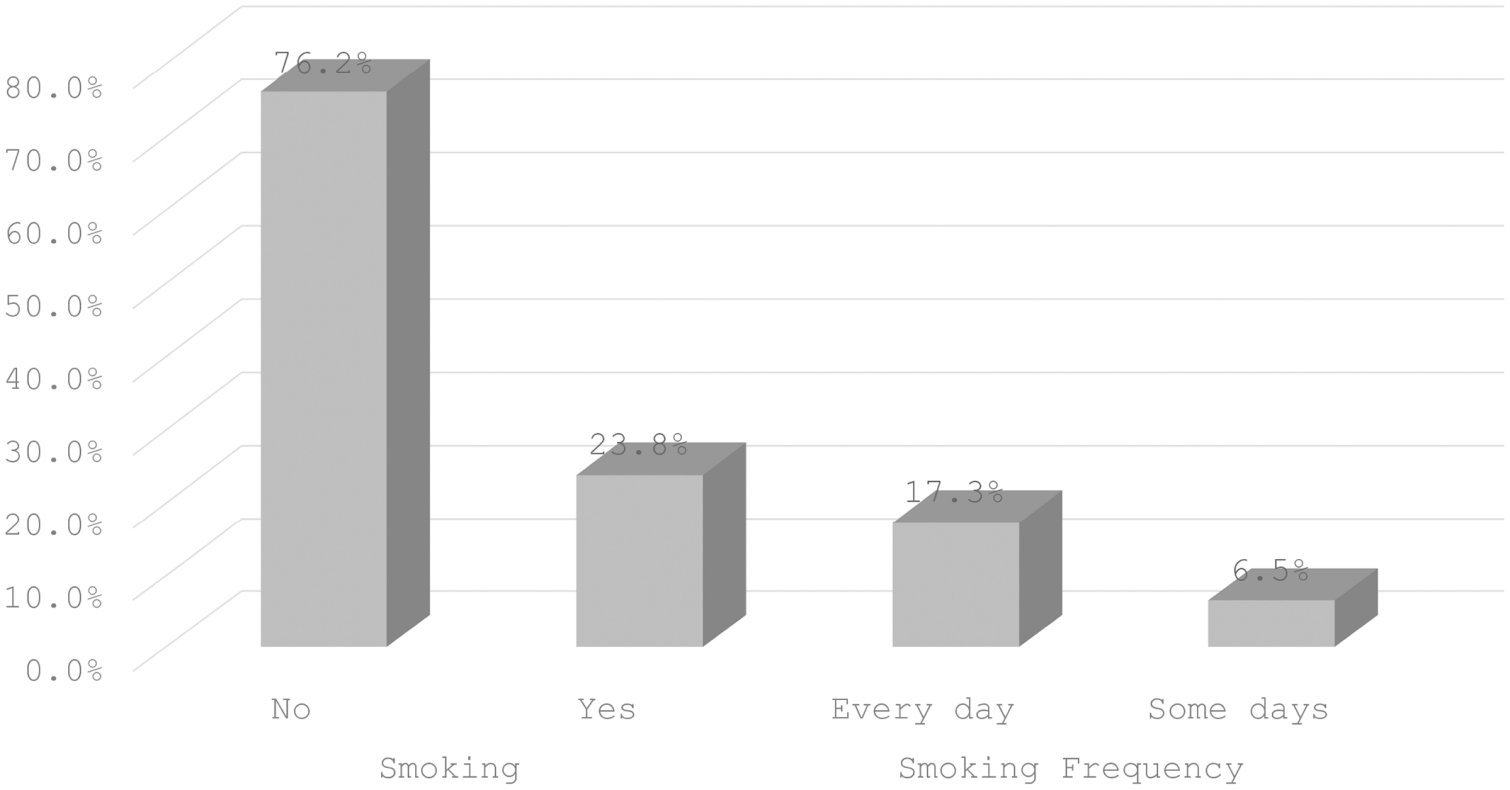
Prevalence of smoking and smoking frequency among people with TB.

The prevalence of cigarette smoking among people with TB is 23.8% (Fig 2). Smoking prevalence among people with TB differed significantly by sex of household head, media exposure‌‌, occupation, alcohol use and attitude to wife beating (Table 2).

**Table 2:** Prevalence of smoking among people with TB (N = 326) in Lesotho, 2023−24.

Parameters		No		Yes		Chi-square	P-value
		N	%	n	%		
Gender	Male	52	42.4	71	57.6	101.94	<0.001^*^
	Female	196	96.7	7	3.3		
Age group	15-19	7	84.5	1	15.5	1.40	0.754
	20-29	24	83.5	5	16.5		
	30-39	68	77.6	20	22.4		
	40 and above	149	74.1	52	25.9		
Marital status	Never in union	31	70.1	13	29.9	1.21	0.765
	Currently in union/living with a woman	163	78.1	46	26.3		
	Formerly in union/living with a woman	54	74.2	19	25.8		
District	Butha-Buthe	10	76.4	3	23.6	5.08	0.662
	Leribe	40	68.6	18	31.4		
	Berea	38	76.6	12	23.4		
	Maseru	110	76.6	33	23.4		
	Mafeteng	13	77.4	4	22.6		
	Mohale’s Hoek	17	90.0	2	10.0		
	Quthing	8	96.3	.3	3.7		
	Qacha’s Nek	5	79.2	1	20.8		
	Mokhotlong	2	72.0	.7	28.0		
	Thaba-Tseka	7	66.6	3	33.4		
Place of residence	Urban	135	77.7	39	22.3	0.41	0.640
	Rural	114	74.4	39	25.6		
Educational level	No education	6	36.2	10	63.8	18.75	0.051
	Primary	87	69.8	38	30.2		
	Secondary	121	82.6	26	17.4		
	Higher	34	88.3	4	11.7		
Religion	Roman Catholic	114	81.8	25	18.2	10.57	0.106
	Protestant	52	63.1	31	36.9		
	Pentecostal/other christians	74	81.4	17	18.6		
	Other religons	8	62.1	5	37.9		
Ethnicity	Basotho	242	77.0	72	23.0	2.92	0.207
	Other tribes	6	52.4	5.0	47.6		
Sex of household head	Male	150	68.9	68	31.1	15.52	0.001^*^
	Female	98	90.8	10	9.2		
Household size	5 or <5	178	75.9	56	24.1	0.02	0.912
	>5	71	76.8	21	23.2		
Media exposure	No media exposure	23	54.1	19	45.9	10.58	0.002^*^
	Yes	225	79.4	58	20.6		
Owns a mobile phone	No	42	66.8	21	33.2	3.08	0.157
	Yes	206	78.4	57	21.6		
Wealth index	Poorest	37	68.2	17	31.8	6.28	0.373
	Poorer	45	75.9	14	24.1		
	Middle	51	73.0	19	27.0		
	Richer	42	72.7	16	27.3		
	Richest	72	86.6	11	13.4		
Occupation	Not working	58	82.3	12	17.7	19.96	0.010^*^
	Professional/Technical/Managerial	10	86.7	2	13.3		
	Clerical/Sales/Services	69	90.7	7	9.3		
	Agricultural workers	12	49.7	12	50.3		
	Manual workers	99	69.2	44	30.8		
Employment	No	99	80.7	24	19.3	1.82	0.264
	Yes	149	73.4	54	26.6		
Total children	No child	31	67.5	15	32.5	7.07	0.366
	One child	61	77.8	17	22.2		
	2-4 children	132	81.2	30	18.8		
	>4 children	25	62.1	15	37.9		
Covered by health insurance	No	221	77.7	63	22.3	2.22	0.389
	Yes	28	66.0	14	34.0		
Alcohol use	Never/No	106	93.9	7	6.1	24.46	<0.001^*^
	Yes	142	66.8	71	33.2		
Wife beating	No	212	79.9	53	20.1	8.82	0.014^*^
	Yes	36	59.9	24	40.1		
TB-related stigma	No stigma	207	74.7	70	25.3	1.68	0.321
	Stigma	41	84.2	8	15.8		
Depression	Missing data	96	94.7	5	5.3	26.35	<0.001^*^
	No depression	114	71.5	45	28.5		
	Yes, depression	38	58.6	27	41.4		

*Significant at p-value = 0.05.

### Prevalence of smoking frequency among people with TB

The prevalence of daily and occasional smoking among people with TB is 17.3% and 6.5%, respectively (Fig 2). The prevalence‌‌ of smoking frequency among people with TB differed significantly by sex of household head, media exposure, alcohol use and attitude to wife beating (Table 3).

**Table 3:** Prevalence of smoking frequency among people with TB (N = 326) in Lesotho, 2023−24.

Parameter		No		Daily		Occasionally		Chi-square	P-value
		n	%	N	%	n	%		
Gender	Male	52	42.4	55	44.9	16	12.7	105.25	<0.001^*^
	Female	196	96.7	1	0.5	6	2.8		
Age group	15-19	7	84.5	1	15.5			1.79	0.956
	20-29	24	83.5	4	13.2	1	3.3		
	30-39	68	77.6	14	15.7	6	6.8		
	40 and above	149	74.1	37	18.7	14	7.2		
Marital status	Never in union	31	70.1	7	16.1	6	13.8	3.96	0.689
	Currently in union/living with a woman	163	78.1	35	16.8	11	5.1		
	Formerly in union/living with a woman	54	74.2	14	19.4	5	6.4		
District	Butha-Buthe	10	76.4	1	11.4	2	2.2	16.74	0.268
	Leribe	40	68.6	11	19.5	7	11.8		
	Berea	38	76.6	8	16.3	4	7.1		
	Maseru	110	76.6	30	20.8	4	2.6		
	Mafeteng	13	77.4	1	6.7	3	15.9		
	Mohale’s Hoek	17	90.0	2	10.0				
	Quthing	8	96.3	0	3.7				
	Qacha’s Nek	5	79.2	0	4.7	1	16.1		
	Mokhotlong	2	72.0	1	28.0				
	Thaba-Tseka	7	66.6	1	13.7	2	19.7		
Place of residence	Urban	135	77.7	30	17.1	9	5.1	1.02	0.729
	Rural	114	74.4	27	17.5	12	8.1		
Educational level	No education	6	36.2	7	45.2	3	18.6	20.33	0.112
	Primary	87	69.8	25	20.1	13	10.1		
	Secondary	121	82.6	20	13.4	6	4.0		
	Higher	34	88.3	4	11.7				
Religion	Roman Catholic	114	81.8	19	13.5	7	4.7	14.03	0.140
	Protestant	52	63.1	24	28.9	7	8.0		
	Pentecostal/other christians	74	81.4	9	10.1	8	8.5		
	Other religons	8	62.1	4	34.4	0	3.5		
Ethnicity	Basotho	242	77.0	52	16.6	20	6.4	3.17	0.320
	Other tribes	6	52.4	4	38.0	1	9.5		
Sex of household head	Male	150	68.9	50	23.0	18	8.1	15.75	0.003^*^
	Female	98	90.8	6	5.8	4	3.4		
Household size	≤5	178	75.9	40	17.2	16	6.8	0.10	0.957
	>5	71	76.8	16	17.4	5	5.8		
Media exposure	No media exposure	23	54.1	15	35.1	5	10.7	10.87	0.007^*^
	Yes	225	79.4	42	14.6	17	5.9		
Owns a mobile phone	No	42	66.8	18	28.9	3	4.3	6.25	0.121
	Yes	206	78.4	38	14.5	17	7.1		
Wealth index	Poorest	37	68.2	15	28.6	2	3.2	12.63	0.320
	Poorer	45	75.9	10	17.4	4	6.7		
	Middle	51	73.0	11	16.3	8	10.7		
	Richer	42	72.7	9	15.8	7	11.5		
	Richest	72	86.6	10	11.8	1	1.7		
Occupation	Not working	58	82.3	7	10.4	5	7.4	21.19	0.061
	Professional/Technical/Managerial	10	86.7	2	13.3				
	Clerical/Sales/Services	69	90.7	5	7.0	2	2.3		
	Agricultural workers	12	49.7	9	36.3	3	14.1		
	Manual workers	99	69.2	33	23.2	11	7.6		
Employment	No	99	80.7	15	11.9	9	7.4	3.32	0.322
	Yes	149	73.4	42	20.5	12	6.0		
Total children	No child	31	67.5	8	18.0	7	14.6	11.30	0.405
	One child	61	77.8	12	15.9	5	6.3		
	2-4 children	132	81.2	23	14.1	8	4.7		
	>4 children	25	62.1	13	32.5	2	5.4		
Covered by health insurance	No	221	77.7	44	15.3	20	7.0	5.24	0.299
	Yes	28	66.0	13	30.7	1	3.3		
Alcohol use	Never/No	106	93.9	3	2.2	4	3.9	25.92	<0.001^*^
	Yes	142	66.8	54	25.3	17	7.9		
Wife beating	No	212	79.9	40	14.9	14	5.2	9.09	0.047^*^
	Yes	36	59.9	17	27.6	8	12.4		
TB-related stigma	No stigma	207	74.7	52	18.7	18	6.5	2.24	0.483
	Stigma	41	84.2	4	9.1	3	6.7		
Depression	Missing data	96	94.7	1	0.5	5	4.8	30.53	<0.001^*^
	No depression	114	71.5	37	23.4	8	5.1		
	Yes, depression	38	58.6	19	28.7	7	12.7		

*Significant at p-value = 0.05.

### Determinants of smoking prevalence among people with TB

Being male (AOR = 107.48, 95%CI:22.65-510.03, p < 0.001), no media exposure (AOR = 3.65, 95%CI:1.43-9.30, p = 0.007), and alcohol use (AOR = 6.98, 95%CI:2.21-22.05, p = 0.001) increased the odds of smoking among PWTB (Table 4). Depression did not significantly influence smoking status of people with TB.

**Table 4:** Determinants of smoking status among people with TB in Lesotho, 2023−24.

Parameters		OR	95% Confidence Interval for OR			AOR	95% Confidence Interval for OR		
			Lower	Upper	Sig.		Lower	Upper	Sig.
	(Intercept)	0.03	0.00	0.17	0.000	0.01	0.00	0.06	<0.001^*^
Gender	Male	89.78	17.46	461.70	0.000	107.48	22.65	510.03	<0.001^*^
	Female	1.00				1.00			
Sex of household head	Male	1.42	0.40	5.11	0.586				
	Female	1.00							
Media exposure	No	3.49	1.26	9.68	0.017	3.65	1.43	9.30	0.007^*^
	Yes	1.00				1.00			
Occupation	Not working	0.70	0.22	2.18	0.531				
	Professional/Technical/Managerial	0.54	0.08	3.79	0.536				
	Clerical/Sales/Services	0.38	0.09	1.67	0.201				
	Agricultural workers	1.33	0.44	3.99	0.614				
	Manual workers	1.00							
Alcohol use	Yes	7.87	2.42	25.57	0.001	6.98	2.21	22.05	0.001^*^
	Never/No	1.00				1.00			
Wife beating Depression	No	0.51	0.22	1.14	0.101				
	Yes	1.00							
	Missing data	7.84	1.18	52.27	0.034	5.99	0.94	38.08	0.058
	Yes	1.44	0.57	3.69	0.440	1.86	0.73	4.73	0.192
	No	1.00				1.00			

*Significant at p-value < 0.05; OR = Odd ratio; AOR = Adjusted odd ratio.

### Determinants of daily smoking among people with TB

Being male (AOR = 211.28, 95%CI:25.86-1725.91, p < 0.001), no media exposure (AOR = 3.95, 95%CI:1.27-12.26, p = 0.018), and alcohol use (AOR = 15.10, 95%CI:3.36-67.89, p < 0.001) increased the odds of daily smoking among PWTB (Table 5).

**Table 5:** Determinants of daily smoking among people with TB in Lesotho, 2023−24.

Parameters		OR	95% Confidence Interval for OR			Exp(B)	95% Confidence Interval for OR		
			Lower	Upper	Sig.		Lower	Upper	Sig.
	(Intercept)	0.01	0.00	0.14	0.001	0.01	0.00	0.05	<0.001^*^
Gender	Male	204.84	25.99	1,614.39	0.000	211.28	25.86	1,725.91	<0.001^*^
	Female	1.00				1.00			
Sex of household head	Male	1.16	0.22	6.04	0.855				
	Female	1.00							
Media exposure	No	3.63	1.13	11.67	0.031	3.95	1.27	12.26	0.018^*^
	Yes	1.00				1.00			
Alcohol use	Never/No	16.74	3.64	77.02	0.000	15.10	3.36	67.89	<0.001^*^
	Yes	1.00				1.00			
Wife beating	No	0.52	0.18	1.49	0.219				
	Yes	1.00							
Depression	Yes	1.28	0.45	3.64	0.635	1.44	0.47	4.39	0.516
	Missing data	1.42	0.09	23.60	0.805	1.29	0.07	22.35	0.861
	No	1.00				1.00			

*Significant at p-value < 0.05; OR = Odd ratio; AOR = Adjusted odd ratio.

### Determinants of occasional smoking among people with TB

Being male (AOR = 39.98, 95%CI:4.76-335.88, p = 0.001) increased the odds of occasional smoking among PWTB (Table 6).

**Table 6:** Determinants of occasional smoking among people with TB in Lesotho, 2023−24.

Frequency currently smokes tobacco		OR	95% Confidence Interval for OR			AOR	95% Confidence Interval for AOR		
			Lower	Upper	Sig.		Lower	Upper	Sig.
	(Intercept)	0.01	0.00	0.13	0.001	0.01	0.00	0.04	<0.001^*^
Gender	Male	33.51	3.73	300.65	0.002	39.98	4.76	335.85	0.001^*^
	Female	1.00				1.00			
Sex of household head	Male	1.61	0.37	6.96	0.524				
	Female	1.00							
Media exposure	No	2.67	0.75	9.46	0.128	3.12	0.91	10.74	0.070
	Yes	1.00				1.00			
Alcohol use	Never/No	3.33	0.84	13.20	0.086	2.83	0.74	10.85	0.128
	Yes	1.00				1.00			
Wife beating Depression	No	0.42	0.13	1.33	0.138				
	Yes	1.00							
	Missing data	12.11	1.04	140.52	0.046	10.74	0.90	128.08	0.060
	Yes	2.87	0.76	10.81	0.119	3.27	0.96	11.22	0.059
	No	1.00				1.00			

*Significant at p-value < 0.05; OR = Odd ratio; AOR = Adjusted odd ratio.

## Discussion

The purpose of this study was to assess the prevalence and determinants of smoking among people with TB in Lesotho. Our findings that the high prevalence of smoking among people with TB is influenced by male gender, media exposure, and alcohol consumption warrant further exploration.

This study found a much higher prevalence of smoking among people with TB than the 18% smoking prevalence in Lesotho’s general population [4]. Since smoking behaviour and impairs immunity and increased the chances of developing TB [17], the high prevalence of TB patient smokers might explain the increasing number of TB cases attributable to smoking in Lesotho [2]. The smoking prevalence in the current study is consistent with evidence from Bangladesh [44], but higher than the findings from other studies in India, Iran, Pakistan, Uganda and Ethiopia [19,25,29,44,45]. In contrast, our finding is lower than the prevalence from prior studies from Gabon, Spain, Brazil, Georgia, Bangladesh, Jordan, Uganda, South Africa, and Botswana [8,11,18,20,24,26–28,30,32,44]. The inter-country differences could be due to baseline differences in the smoking habits of the general population from which the TB patients’ subpopulations were derived.

Our finding of high TB patient smokers might explain the increasing number of TB cases attributable to smoking in Lesotho [2]. The high prevalence of TB patient smokers may be due to limited smoking control measures or a higher addiction to nicotine among people with TB. Notwithstanding signing the World Health Organization’s Framework Convention on Tobacco Control, the Government of Lesotho lacks tobacco packaging regulations and regulations on tobacco advertising, promotion and sponsorship. While a restricting or eliminating marketing is key to tobacco control success, Lesotho’s government has not implemented any component of the direct and indirect bans on TAPS. Similarly, the weak tobacco tax policies incentivize tobacco users by making tobacco products affordable. Additionally, the Government of Lesotho need to integrate routine tobacco use screening and smoking cessation counselling into TB care to help people with TB quit smoking.

Consistent with evidence from previous studies [11,17,28,30–33], our study finding revealed a higher likelihood of smoking, daily smoking and occasional smoking among males with TB, In the current study, 57.6% of men with TB were smokers, compared to 39.2% of males in the general population. Contrastingly, we found that 3.3% of women with TB smoked compared to 0.4% in the general population. The lower prevalence of smoking among women with TB in this study is not unsurprising given the cultural situation of women in most sub-Saharan African settings. Deep social norms and taboos discourage women from smoking and cigarette smoking is deemed an inappropriate behaviour for women, while smoking among men is acceptable, and represents a symbol of status and social power [33,46]. The significant association of smoking with male gender might account for the gender differences in TB prevalence with more male TB cases than females [11,17]. Additionally, the gender disparity may also result from endogenous hormonal variations, particularly the influence of female sex hormones, may enhance women’s immune responses to cytotoxic challenges [3]. Our finding highlights a need for gender-sensitive tobacco use screening, assessment, and cessation counselling tailored to men with TB.

We also found that a lack of media exposure predicted smoking among people with TB in Lesotho. People with TB who lacked media exposure were almost four times more likely to smoke. A lack of media exposure also increased the odds of daily smoking. Our findings imply a beneficial influence of media exposure on reducing smoking prevalence among people with TB. Although no prior study has assessed how exposure to tobacco-related media influences smoking behaviour among people with TB, evidence indicates that high media exposure lowers the risk of cigarette smoking among adults in the general population [47,48]. Exposure to anti-smoking messages was associated with greater risk perceptions of smoking, which could facilitate prevention of smoking initiation and intention to quit [49]. Nevertheless, tobacco control in Africa, including Lesotho, is constrained by limited use of best-practice anti-tobacco media campaign, the competition to use the media to push for or against tobacco control, a lack of long-term plan for anti-tobacco media, inadequate funding for mass media interventions, and low involvement of target beneficiaries in the process of developing anti-tobacco media products [50,51].

In this study, alcohol consumption increased the likelihood of smoking and daily smoking among people with TB. Similarly, alcohol increased the odd of smoking among people with TB in previous studies [11,17,33,34]. Our finding mirrors the influence of alcohol use on smoking in the general population reported in other African studies [52,53]. Alcohol consumption increases the urge and impulse to smoke by disrupting cognitive functions, weakening self-control or self-regulation, interacting with the brain nicotinic receptors and activating dopamine release [54]. In the current study, about 33% of people with TB using alcohol smoked compared to about 6% of people with TB, who do not use alcohol. This evidence will enable the National TB Program (NTP) in Lesotho to target TB patients, who use alcohol, with smoking cessation interventions and initiatives to reduce alcohol use. It is essential for the NTP to integrate management of substance use disorder into routine TB services.

Surprisingly, depression did not increase the likelihood of smoking and smoking frequency among people with TB in this study. Contrastingly, prior studies show that depressive symptoms increased the risk of smoking among TB patients, while smoking predisposed TB patients to depression [30,55]. Long TB treatment period can lead to mental and emotional problems, necessitating smoking. Smokers, including people with TB, use nicotine to medicate their depressed mood because of the reinforcing effects of nicotine’s mood-altering characteristics [56]. Nicotine, the main content of tobacco, causes the release of dopamine, which improves mood and reduces mental stress [30,56]. Yet, our finding should be interpreted with caution, given that about 31% of people with TB in this study had no mental health data, since the LDHS 2023−24 collected mental health data from 50% of the household sample [40].

The main strength of this study was going beyond the social characteristics of respondents to explore the influence of TB-related stigma and mental health status of people with TB on smoking status. Although the LDHS 2023−24 was designed to be highly representative, using a robust design, including a large sample size and stratification by district and area type, the current study may not be representative data on smoking prevalence among TB patients in Lesotho. We have analyzed the secondary data that we had no control over from the point of data collection. Moreover, smoking status capture is historically low in such datasets due to social desirability bias. Even so, our study provides useful insights into the prevalence and determinants of smoking among people with TB without such claims of generalizability and representation. While smoking intensity might influence smoking status of people with TB [33], it was not possible for this study to test that hypothesis, since the LDHS 2023−24 dataset lacked data on quantity of tobacco consumed daily. Furthermore, being a cross-sectional survey, the study can only show association between smoking and the determinants and cannot establish causality.

## Conclusion

The purpose of this study was to determine the prevalence of smoking among people with TB and its associated factors in Lesotho. The prevalence of smoking among people with TB is high in Lesotho. Male gender, a lack of media exposure, and alcohol use are risk factors for smoking among people with TB in Lesotho. These findings underscore the importance of gender-sensitive tobacco use prevention and control interventions, an inclusive and adequately funded long-term plan for best-practice anti-tobacco media campaign, and integrating management of substance use disorder into routine TB care to address the intersection of alcohol use and smoking in the social epidemiology of TB. Policymakers and health system practitioners should incorporate the identified social factors into the National Strategy for TB Control Program to ensure effective implementation of interventions to reduce smoking initiation and increase smoking cessation among people with TB in Lesotho.

## Abbreviations

DALY: Disability-adjusted life years
DHS: Demographic and Health Survey
EA: Enumeration Area
GDP: Gross Domestic Product
LDHS: Lesotho Demographic and Health Survey
LMICs: Low- and Middle-Income Countries
PHC: Primary Health Care
PHQ: Patient Health Questionnaire
PSU: Primary Sampling Unit
SPSS: Statistical Package for Social Sciences
SSA: Sub-Saharan Africa
TB: Tuberculosis
VIF: Variable Inflation Factor
